# Optimizing grout formulations for post-tensioning using pozzolanic and filler blends

**DOI:** 10.1038/s41598-025-30147-6

**Published:** 2025-12-14

**Authors:** Sarah Ibrahim, Ayman Shamseldein, Hesham Sokairge, Hany Elshafie

**Affiliations:** Structural Engineering Department, Faculty of Engineering, Ain Shams University, Cairo, Egypt

**Keywords:** Post tension, High performance grout, Durability, Mechanical properties, Limestone powder, Engineering, Materials science

## Abstract

The use of post-tensioning (PT) technology has grown rapidly due to the increasing demand for longer spans and reduced section depths in concrete structures. However, conventional grout used in bonded PT systems often suffers from bleeding and segregation, leading to void formation, tendon corrosion, and reduced durability. To overcome these issues, high-performance grout (HPG) mixes are typically produced using supplementary cementitious materials (SCMs) such as silica fume and fly ash. Although effective, these materials are expensive and not always readily available. This study investigates the feasibility of using limestone powder as a cost-effective and locally available alternative to traditional SCMs. Thirteen grout mixes were developed with varying water-to-cementitious material ratios (0.27–0.45) and partial replacements of cement by limestone powder (25% and 35%), fly ash (25% and 35%), and silica fume (2–10%).Experimental results demonstrated that increasing limestone powder content reduced bleeding from 1.4% to 0.5% due to its filler effect, but also increased efflux time beyond 50 s and reduced compressive strength from 35 MPa to 24 MPa. In contrast, silica fume-based mixes exhibited superior performance, achieving compressive strengths of 40–55 MPa, bleeding rates below 0.2%, and efflux times of 23–26 s, fully complying with ACI/PTI HPG standards. Fly ash improved workability and long-term strength but did not fully control bleeding. Overall, silica fume provided the best balance of strength, flowability, and durability, while limestone powder served as a low-cost, non-reactive filler capable of moderately enhancing bleeding resistance. The findings indicate that limestone powder can be utilized as a partial substitute for costly SCMs in resource-limited regions, contributing to more sustainable and economical PT grout formulations.

## Introduction

Concrete is the most used construction material globally^1,2^. Since concrete is strong in compression but weak in tension, it is prone to cracking when exposed to high tensile forces. To overcome this limitation, engineers developed techniques such as prestressing and post-tensioning, which enhance the tensile strength of concrete, allowing for more durable and efficient structures^3^. The introduction of prestressed concrete enabled engineers to construct structures capable of supporting heavier loads and spanning longer distances with minimal cracking or deflection^4^. Post-tensioning can be applied using two main systems: bonded and unbonded. These differ based on whether grout is used to surround the tendons or not. The grout in bonded post-tensioning systems plays a vital role in transferring stress, providing corrosion protection to tendons, and maintaining overall structural integrity^5,6^. However, improper grout selection or performance issues such as bleeding can lead to serious durability problems, including tendon corrosion. Key properties of high-quality post-tensioning (PT) grouts include fluidity, retention of fluidity over time, yield stress, apparent viscosity, resistance to bleeding, setting time, dimensional stability, and compressive strength. The grout must be sufficiently fluid to completely fill even the smallest gaps between the tendons and the duct, maintaining this fluidity throughout the grouting operation. A reduction in fluidity increases the pumping pressure required to place the grout^7^. Fluidity and its retention are commonly evaluated using tests such as the Marsh cone test, which measures the efflux time, and the spread test, which determines the diameter of the spread at various intervals after mixing. Bleed resistance is a crucial parameter that defines the quality of PT grouts^8–10^ as it prevents the separation of water from the solid grout components (segregation). Several testing methods have been developed to assess bleeding under conditions that simulate actual field scenarios, including the standard bleed test, wick-induced bleed test, inclined tube bleed test^11^, and pressure-induced bleed test^12^. Another issue arising from segregation is the formation of soft grout^8,9^. Excessive bleeding can cause the solid particles to separate, resulting in soft grout formation. While bleed water may be reabsorbed during hydration, soft grout typically has a lower pH, retains moisture, and can be highly corrosive to tendons. The presence of soft grout near corroded tendons has been linked to grout segregation^8,13^. A comprehensive study conducted by Kamalakannan et al.^10^ investigated the performance of commercially available grout products for PT systems. Findings revealed that many of the grout products commonly used in developing countries failed to meet both international standards and the manufacturers’ claimed specifications. Moreover, the performance of the grout was significantly affected by factors such as mixing speed, ambient temperature, and material fineness. The study showed the critical importance of evaluating grout behavior under realistic field conditions to ensure void-free and durable PT systems, particularly in regions where grouting technology is still developing. Therefore, researches was conducted to focus on optimizing grout mixes to ensure they meet the demanding performance criteria required for modern post-tensioned systems^14^. In 2021, Mohan et al.^15^ conducted a comprehensive three-phase study aimed at developing high-performance, pre-blended cementitious grouts with enhanced properties. Their investigation focused on overcoming common deficiencies in commercial grouts, particularly poor fluidity and resistance to segregation. In their study, a range of grout mixtures was carried out using varying proportions of ordinary Portland cement (OPC), fly ash, and chemical admixtures. Through iterative testing and performance-based selection across the three phases, an optimal grout formulation was developed. This final mix, comprising 52% OPC and 48% fly ash by volume, along with optimized admixture dosages, was industrially pre-blended and shown to meet performance specifications such as EN447^11^ and PTI M55^16^. In addition to improving the performance of grout, supplementary cementitious materials also play a crucial role in reducing the reliance on cement, thereby contributing to a significant reduction in carbon emissions. The UN Intergovernmental Panel on Climate Change in COP27^17^ held in Sharm el-Sheikh recommended that CO2 emissions must be reduced with 45% by 2030, to enable compliance with the criterion of not exceeding 2 °C temperature by 2050.Therfore, the use of supplementary cementitious materials as a replacement of cement is very important. In 2023, Mohan et al.^18^ compared the performance of a novel pre-blended grout with a commonly used site-batched grout by examining their respective mix compositions. The preblended grouts were formulated using 935 kg/m^3^ of ordinary Portland cement (OPC) and a substantial quantity of Class F fly ash 311 kg/m^3^ each from two sources resulting in a total of 622 kg/m^3^ of fly ash. In contrast, the site batched grout mix contained a higher amount of OPC (1300 kg/m^3^) and no supplementary cementitious materials. The water content in preblended grout was significantly lower (420 kg/m^3^) compared to 585 kg/m^3^ in the site batched grout mix. Additionally, preblended incorporated several chemical admixtures to enhance its performance, including a high-range water reducer (1.43 kg/m^3^), a viscosity-modifying admixture (0.63 kg/m^3^), and a shrinkage-reducing admixture (31.7 kg/m^3^). On the other hand, the SBG mix included only a plasticized expansive admixture (5.85 kg/m^3^) and lacked any advanced rheology- or shrinkage-controlling agents. The preblended grout mix design aimed to improve fluidity retention, minimize bleeding, reduce shrinkage, and enhance overall grout stability under field conditions. These performance improvements were validated through comprehensive testing, with the preblended grout satisfying all the proposed specifications for post-tensioning applications. In contrast, the site batched grout mix exhibited deficiencies in key areas such as bleeding resistance and volumetric stability, underscoring the superiority of the engineered TFG mixes for durable corrosion protection of PT tendons. In 2024, Ibrahim et al.^19^ published a comprehensive review article on high-performance grouts, highlighting the current advancements and challenges in the field. The article recommended that future research efforts should focus on the development of sustainable grout formulations by incorporating locally available and environmentally friendly materials to reduce costs and carbon footprint. They also emphasized the need to explore innovative admixtures, such as nanomaterials and polymers, to enhance mechanical properties, durability, and resistance to segregation and bleeding. Furthermore, Ibrahim et al. stressed the importance of investigating the long-term performance and shelf life of these novel grout formulations under varying environmental conditions to ensure their reliability in practical applications. The review called for multidisciplinary approaches combining material science, structural engineering, and environmental sustainability to drive the next generation of high-performance grouts. Although silica fume and fly ash have been widely used to produce high-performance grouts, their high cost, limited availability, and variability in quality have motivated the search for alternative materials, particularly in developing regions. Among potential substitutes, limestone powder (LP) has attracted attention due to its fine particle size, chemical stability, and widespread local availability. Previous studies have shown that limestone can act as a physical filler that enhances particle packing, reduces capillary porosity, and improves the early-age performance of cementitious systems (Sua-iam and Makul^30^; Randell et al.^13^. However, unlike pozzolanic materials such as silica fume or fly ash, limestone powder is largely inert and does not participate in hydration reactions, which may lead to a reduction in long-term strength if used excessively. Therefore, an optimal replacement level is essential to balance improvements in workability and bleeding resistance against possible strength reductions.Based on these insights, this study investigates the comparative performance of limestone powder, silica fume, and fly ash in grout mixes designed for post-tensioning applications. The aim is to evaluate whether locally available limestone powder can serve as a cost-effective supplementary material capable of partially replacing expensive SCMs while maintaining compliance with ACI/PTI performance criteria. The findings presented in this work demonstrate that limestone powder can reduce bleeding due to its filler effect but increases efflux time and may reduce compressive strength, whereas silica fume provides the best balance of mechanical and durability properties. This comparison provides a clear basis for developing sustainable and economical high-performance grouts in resource-limited contexts. Therefore, limestone powder was selected as a potential replacement for silica fume and fly ash, which are widely used in grout production but are often associated with high material costs and limited availability. Before conducting the experimental program, it was hypothesized that incorporating limestone powder could improve the bleeding resistance of grout due to its physical filler effect and enhanced particle packing density. The fine limestone particles were expected to fill voids between cement grains, reducing capillary water channels and, consequently, limiting water migration and segregation. This mechanism was anticipated to lower bleeding while maintaining acceptable flow characteristics if used in moderate proportions. However, since limestone powder is largely inert and lacks pozzolanic reactivity, it was also expected that excessive replacement levels might reduce compressive strength due to dilution of the cementitious binder. Thus, the experimental program was designed to verify whether limestone powder could serve as a cost-effective, partially substitutive material that balances reduced bleeding with adequate flowability and mechanical performance. Therefore, this research was conducted with the aim of developing a grout mix design that meets specified performance requirements while remaining cost-effective. A total of thirteen grout mixtures were developed by varying the water-to-cementitious material ratios (W/Cm) and adjusting the proportions of cement, fly ash, silica fume, limestone powder, expansive admixtures, and high-range water-reducing agents. The objective was to determine the optimal formulations that exhibit key high-performance grout (HPG) characteristics, such as enhanced compressive strength, adequate flowability, minimized bleeding, and stable dimensional behavior.

## Research significance

Identifying research gaps and defining a focused direction often require substantial experience. To support this process, a bibliometric analysis was performed using keywords relevant to post-tensioning grout in the Scopus database. The analysis, visualized through VOSviewer, revealed that although grout plays a critical role in tendon protection and long-term durability, it remains less explored compared to broader concrete research areas.A notable gap was identified in studies evaluating low-cost or low-carbon supplementary materials, particularly limestone powder, as partial substitutes for traditional pozzolanic additives such as silica fume and fly ash. Most previous work concentrated on enhancing grout performance using high-cost SCMs, leaving limited attention to affordable alternatives suitable for developing regions.Therefore, this study aims to address these gaps by experimentally investigating the feasibility of limestone powder as a sustainable, locally available additive to improve bleeding resistance and dimensional stability of grout while maintaining adequate flowability and strength. The insights gained are expected to contribute to developing cost-effective high-performance grout formulations for post-tensioning applications.

## Experimental program—materials and methodology

The experimental program involved testing thirteen different grout mixes. The process began with the preparation and testing of a conventional grout mix commonly used in situ, following the ACI/PTI 320 standard^11^. The initial tests evaluated compressive strength, flowability, and bleeding characteristics. Subsequently, the mix was optimized by incorporating various proportions of silica fume, fly ash, expansive agents, and high-range water-reducing admixtures. Once the optimal mix that satisfied the required standards was identified, limestone powder was introduced in later trials as a cost-effective substitute for more expensive additives such as silica fume and fly ash. Finally, only the mixes that met the standard requirements for compressive strength, bleeding, and flowability were subjected to further testing to assess their dimensional stability.

### Material properties

In this study, Cement type CEM II/A-P 42.5 N, compliant with the EN 197-1 standards^20^, was selected as the base binder for all grout mixes. CEM II/A-P is a Portland composite cement that contains a blend of Portland cement clinker and basalt powder as pozzolanic material. This type of cement is commonly used in construction due to its balance of performance, cost-effectiveness, and sustainability. All mixes were prepared using potable water, which meets the standards for water quality in concrete mixing. Potable water ensures that the mix is free of impurities that might affect the hydration process and the overall performance of the grout. To minimize shrinkage and enhance the dimensional stability of the grout, Sika Intraplast Z, a shrinkage-reducing admixture with a density of approximately 1.07 g/cm^3^, was incorporated into the mix. Additionally, Sika ViscoCrete-3425, a high-range water-reducing admixture with a density of 1.06 g/cm^3^, was added to improve fluidity and workability. This admixture allows for reduced water content while maintaining excellent flow characteristics, thereby enhancing compressive strength and facilitating easier pumping and placement. To further improve the grout’s performance, Sika Fume (silica fume) and Sika Fly Ash were used as mineral additives to boost strength, durability, and chemical resistance. Sika Fume is a fine gray powder with a specific gravity of 2.2, while Sika Fly Ash also has a specific gravity of 2.2. Additionally, limestone powder, with a specific gravity of 2.7, was included as a supplementary filler. All material properties were obtained from the respective manufacturer data sheets. The chemical and physical properties of cement, silica fume, fly ash and limestone powder as per the data sheets of manufacturer are shown in Table 1, and Table 2 respectively.

**Table 1:** Chemical properties of binder materials.

Chemical property	Cement	Silica fume	Fly ash	Limestone powder
Silicon dioxide (SiO_2_) %	18–22	~ 90–95	45–55	0.5–2.5
Aluminum oxide (Al_2_O_3_) %	4–6	0.5–1.5	25–30	0.1–0.5
Iron oxide (Fe_2_O_3_) %	2–4	0.2–0.8	6–12	0.1–0.3
Calcium oxide (CaO) %	60–65	0.2–0.8	2–6	95–98
Magnesium oxide (MgO) %	1–2	0.5–1.2	1–3	0.5–1.5
Sulfur trioxide (SO_3_) %	2.5–3.5	0.1–0.5	0.5–1.5	0.01–0.1

**Table 2:** Physical properties of binder materials.

Material	Cement	Silica fume	Fly ash	Limestone powder
Specific gravity	3.15	2.2	2.2	2.7
Particle size (D50)	15 μm	0.15 μm	20 μm	8 μm

### Mix proportions

Table 3 summarizes the material proportions for the thirteen grout mixes used in this study. The experimental program was designed to investigate the influence of three primary factors on grout performance:

1. the water-to-cementitious materials ratio (W/Cm),
2. the type of supplementary cementitious material (SCM), and.
3. the replacement level of cement by each SCM.

The factor levels for the study were selected based on previous research on high-performance grout (Mohan et al.^15^, Khayat et al.^8^ and on preliminary laboratory trials to ensure measurable variations in flowability, bleeding, and compressive strength.

- **W/Cm ratios**: 0.27, 0.30, 0.35, and 0.45 were used to evaluate the influence of water content on workability and bleeding control.
- **SCM type**: Fly ash (FA), silica fume (SF), and limestone powder (LP) were used to compare the effects of pozzolanic and non-pozzolanic additives.
- **Replacement levels**: Fly ash and limestone powder replaced cement by **25% and 35%**, while silica fume replaced cement by **2%**,** 6%**,** and 10%** by weight.

Thirteen mixes were developed and grouped accordingly:

- **Group 1 (M1–M4)**: Conventional grout mixes with varying W/Cm ratios (0.45, 0.35, 0.27) to establish the control baseline and assess the effect of adding a high-range water-reducing admixture (HRWR).
- **Group 2 (M5–M6)**: Fly ash replacement mixes (25% and 35%).
- **Group 3 (M7–M10)**: Limestone powder replacement mixes (25% and 35%) prepared with W/Cm ratios of 0.27 and 0.30.
- **Group 4 (M11–M13)**: Silica fume replacement mixes (2%, 6%, and 10%) at constant W/Cm = 0.27.

Each mix is identified using a systematic code that reflects its composition. The mix code is structured as M[number]–[W/Cm]– [Additive Information], where “M” followed by a number (e.g., M1, M2, etc.) serves as a sequential identifier for the mix. The middle portion of the code represents the water-to-cementitious material ratio (W/Cm), while the final portion denotes the type and proportion of any supplementary cementitious material (SCM) or filler included, such as fly ash (FA), silica fume (SF), or limestone powder (LP). For example, Mix M1-0.45-0 represents a grout with a W/Cm of 0.45 and no additional mineral additives. Mix M5-0.27 H-25FA refers to a grout with a W/Cm of 0.27, containing 25% fly ash as a partial replacement for cement, and includes a high-range water-reducing admixture. The inclusion of the letter “H” in the mix code (as in “0.27H”) indicates that the mix incorporates Sika ViscoCrete-3425, a high-range water-reducing admixture (HRWRA), which was used at a dosage of 1.5% by weight of the total cementitious materials to improve flowability and reduce the water requirement.

**Table 3:** Mix proportions.

Serial	Mix code	W/Cm	Cement	FA	SF	LP	Intraplast Z	ViscoCrete 3425
1	M1-0.45-0	0.45	1	-	-	-	2% of cementitious material	-
2	M2-0.35-0	0.35	1	-	-	-		-
3	M3-0.27-0	0.27	1	-	-	-		-
4	M4-0.27 H-0	0.27	1	-	-	-		1.5% of cementitious material
5	M5-0.27 H-25FA	0.27	0.75	0.25	-	-		
6	M6-0.27 H-35FA	0.27	0.65	0.35	-	-		
7	M7-0.30 H-25LP	0.30	0.75	-	-	0.25		
8	M8-0.30 H-35LP	0.30	0.65	-	-	0.35		
9	M9-0.27 H-25LP	0.27	0.75	-	-	0.25		
10	M10-0.27 H-35LP	0.27	0.65	-	-	0.35		
11	M11-0.27 H-2SF	0.27	0.98	-	0.02	-		
12	M12-0.27 H-6SF	0.27	0.94	-	0.06	-		
13	M13-0.27 H-10SF	0.27	0.90	-	0.1	-		

### Specimen preparation and testing set up

In this study, four key tests were conducted on each grout mix to comprehensively evaluate its mechanical and physical performance characteristics. These tests included compressive strength, flowability, bleeding, and dimensional stability. The compressive strength of the grout was carried out according to ASTM C942^21^, which is the standard test method for evaluating the compressive strength of grouts for preplaced-aggregate concrete in laboratory conditions. For each mix, six cube specimens measuring 50 mm were cast using standardized molds. After demolding at 24 h, the specimens were cured under controlled conditions. Three cubes were tested at 7 days to evaluate early strength development, while the remaining three were tested at 28 days to determine long-term strength. The average values for each age were calculated to ensure representative and reliable results for comparative analysis.

**Fig. 1:**
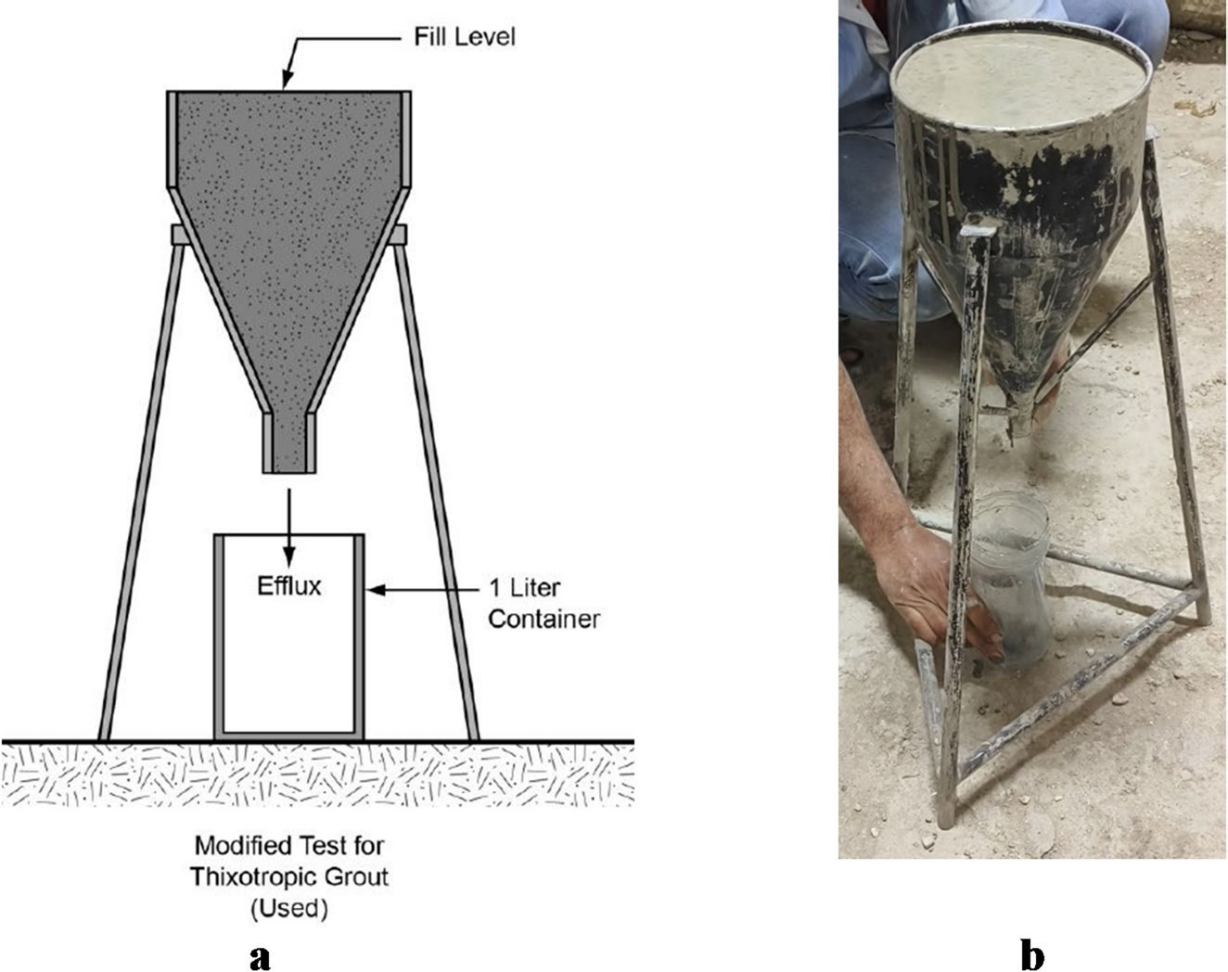
(a) Schematic diagram of the flowability testing device; (b) Photograph of the actual flowability testing setup.

The flowability of the grout was evaluated using the ASTM C939 method^22^, which involves measuring the efflux time it takes for the grout to flow through a standard flow cone. This test is essential for assessing the grout’s ability to fill voids and flow easily around reinforcement without segregation. Proper flowability is particularly critical in applications where pumping or gravity flow is required. A schematic diagram of the flow cone used for testing is shown in Fig. 1a, while Fig. 1b presents an actual photograph of the apparatus. These visual aids help illustrate the setup and ensure clarity in the testing methodology.

**Fig. 2:**
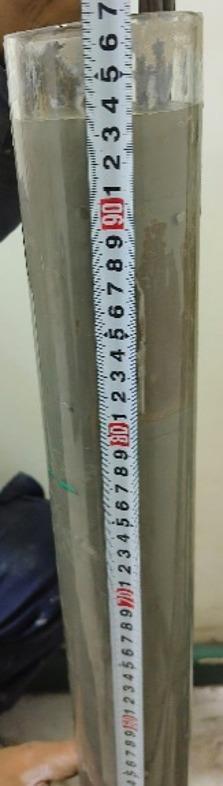
Wick induced bleeding test.

The bleeding test was conducted following ASTM C940^23^, using the wick-induced method. This method evaluates the amount of bleed water that rises to the surface of freshly mixed grout, which is an important factor affecting durability, uniformity, and bond strength. Excessive bleeding can lead to segregation, reduced cohesion, and long-term durability issues. The wick-induced method enhances the sensitivity of bleed water detection and provides a more realistic simulation of field conditions. The test setup is illustrated in Fig. 2.

**Fig. 3:**
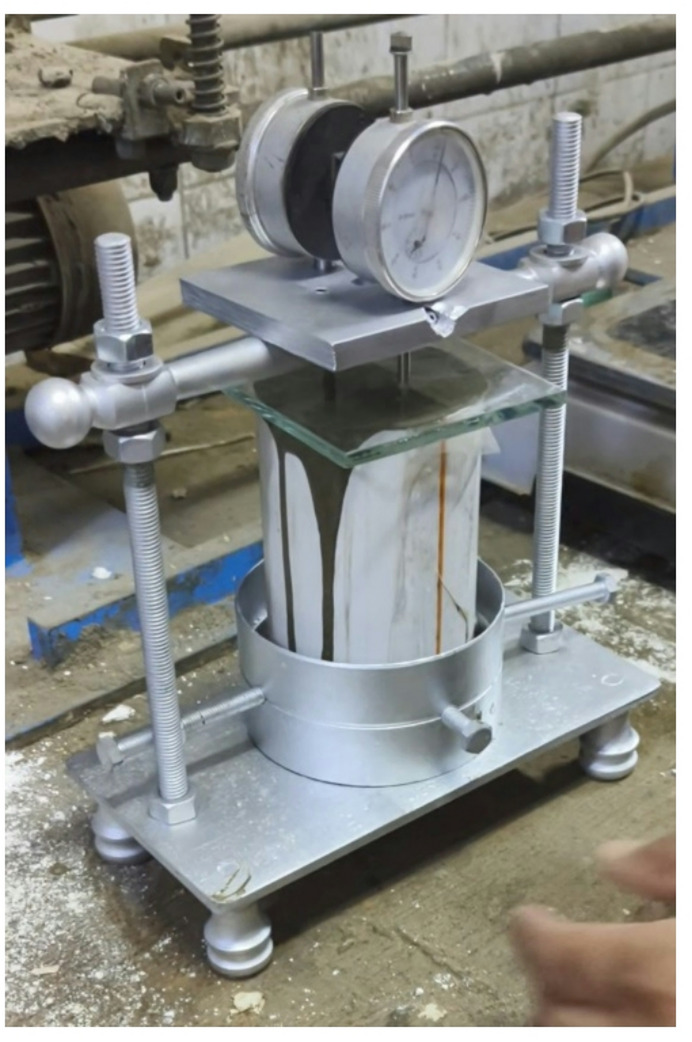
Dimensional stability test.

Lastly, the dimensional stability of the grout, which refers to its tendency to shrink or expand over time, was assessed using the procedure outlined in ASTM C1090^24^. These standard measures the change in height of cylindrical specimens over a specified period, allowing for the evaluation of volume stability. Shrinkage or expansion can significantly affect the performance of grout in structural applications, potentially leading to cracking or loss of contact with embedded components. The device used for this test is shown in Fig. 3. Regular measurements were taken at predetermined intervals to monitor volumetric changes and determine the extent of dimensional stability across different mixes.

## Results and discussion

The performance evaluation of grout mixes in this study focused on three key parameters such as flowability, bleeding, and compressive strength as these represent the most critical and interdependent indicators of high-performance grout behavior according to standards. Flowability determines the grout’s ability to fully penetrate ducts and encapsulate tendons, while bleeding reflects the mixture’s stability and resistance to segregation. Both parameters directly influence compressive strength, since excessive bleeding increases porosity and reduces strength and durability. Therefore, achieving an appropriate balance among these three properties is essential to ensure effective tendon protection and structural integrity in bonded post-tensioning systems. Additional dimensional stability tests were conducted on selected mixes that met standard compliance to further assess volumetric behavior.

**Fig. 4:**
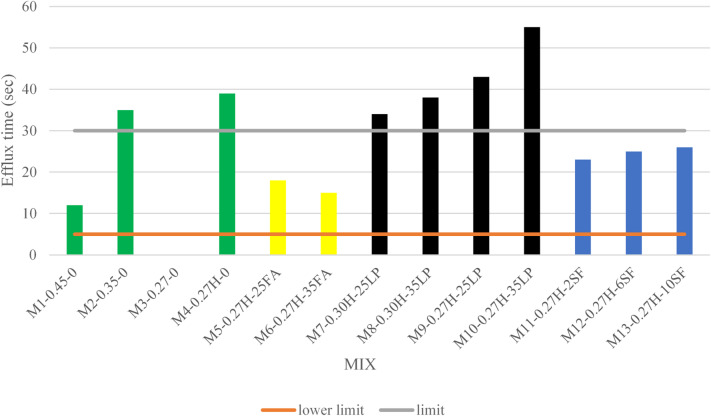
Flowability test results.

**Fig. 5:**
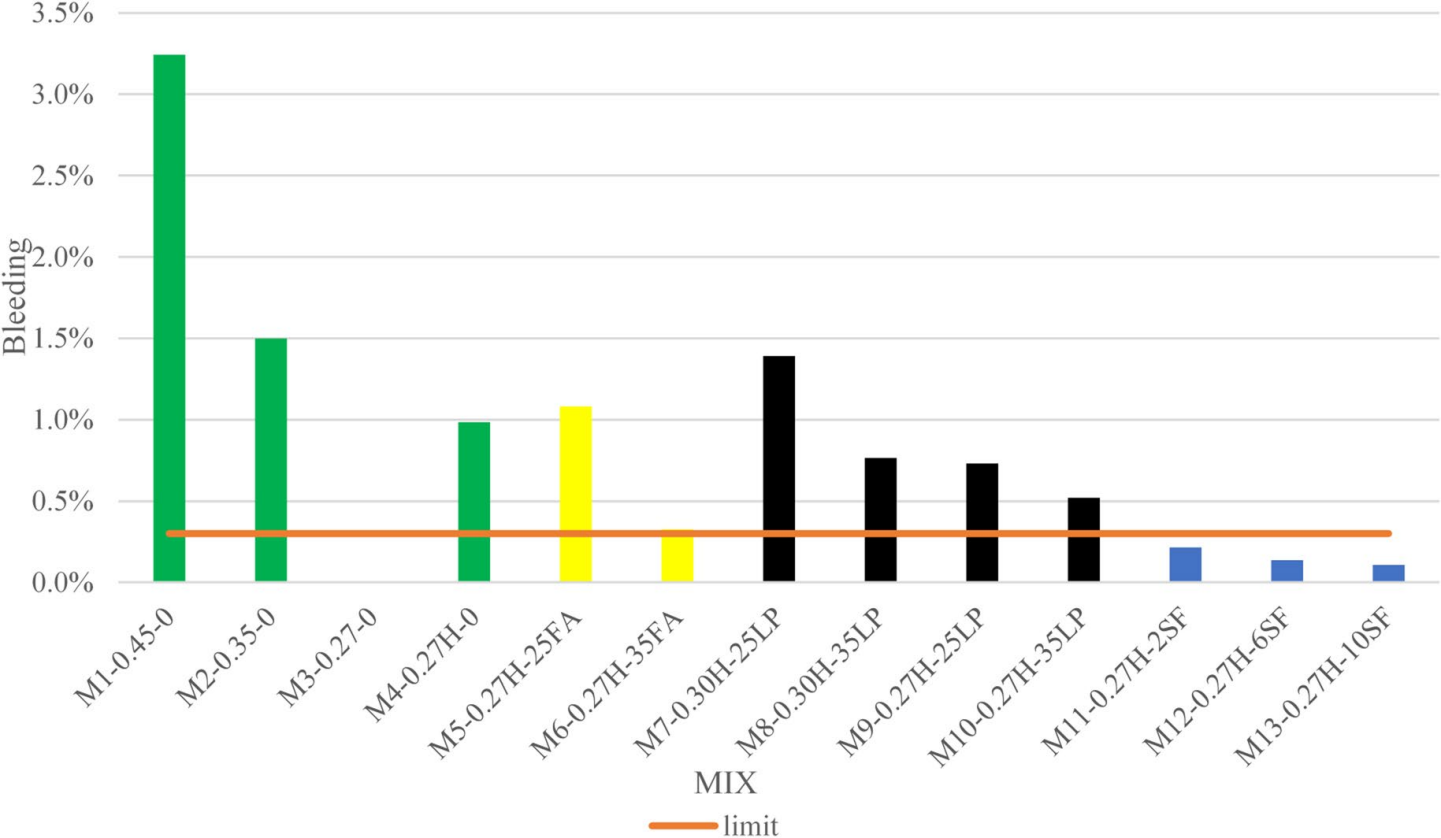
Bleeding test results.

**Fig. 6:**
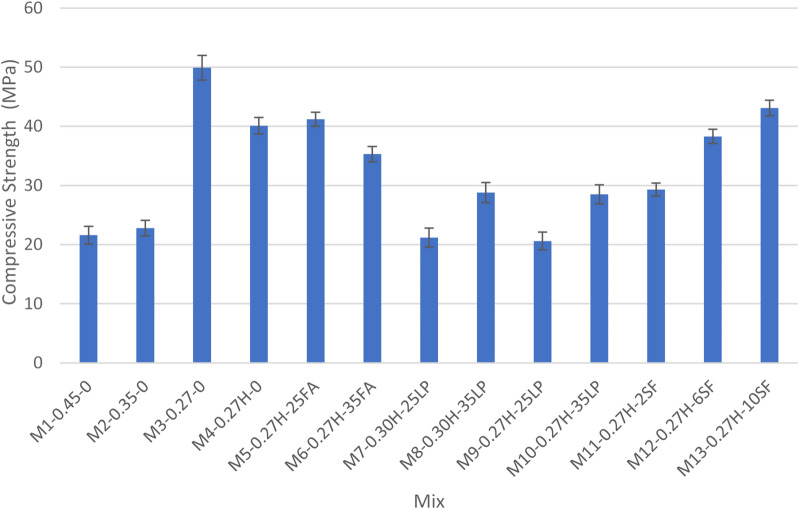
Compressive strength at 7 days test results.

**Fig. 7:**
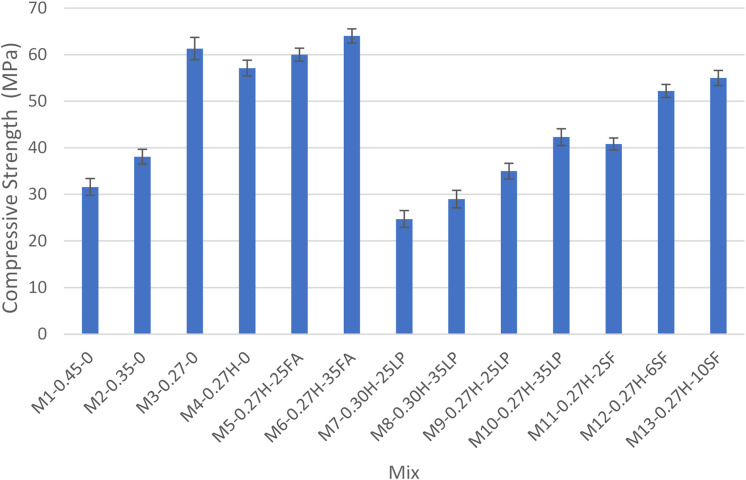
Compressive strength at 28 days test.

**Fig. 8:**
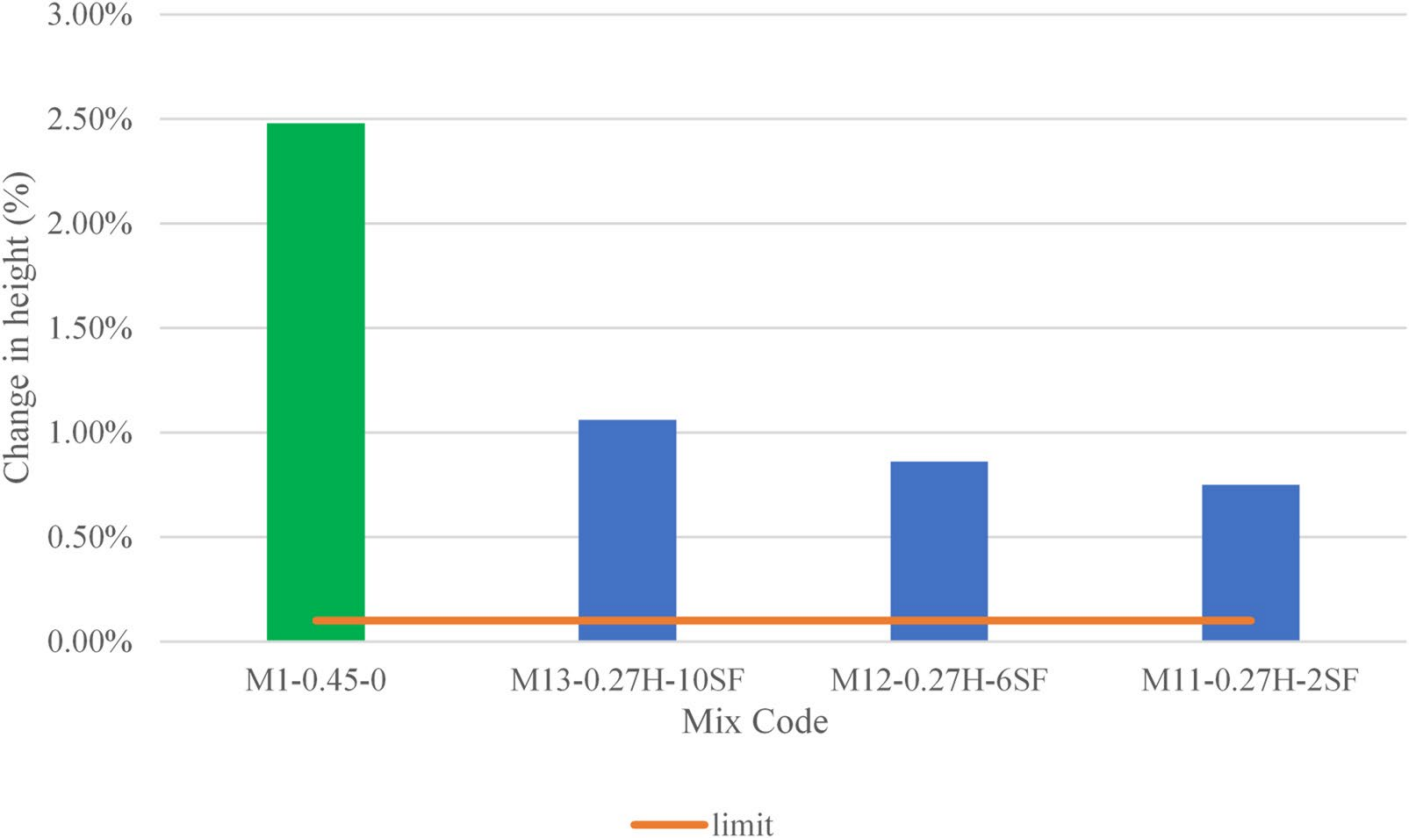
Dimensional stability test results.

A consolidated table summarizing the test results for all grout mixes is presented in Table 4, while Figs. 4, 5, 6, 7 and 8 display the results as bar charts for easier comparison.

**Table 4:** Test results.

Mix code	Efflux time (sec)	Bleeding (%)	Compressive strength at 7 days		Compressive strength at 28 days		Volume change (%)
			Mean (MPa)	Standard deviation (MPa)	Mean (MPa)	Standard deviation (MPa)	
M1-0.45-0	12	3.2%	21.6	± 1.5	31.6	± 1.8	2.48%
M2-0.35-0	35	1.5%	22.8	± 1.3	38.1	± 1.6	-
M3-0.27-0	-	-	49.9	± 2.1	61.3	± 2.4	-
M4-0.27 H-0	39	1%	40.1	± 1.4	57.1	± 1.7	-
M5-0.27 H-25FA	18	1.08%	41.2	± 1.2	60.0	± 1.4	-
M6-0.27 H-35FA	15	0.33%	35.3	± 1.3	64.0	± 1.5	-
M7-0.30 H-25LP	34	1.4%	21.2	± 1.6	24.7	± 1.8	-
M8-0.30 H-35LP	38	0.8%	28.8	± 1.7	29.0	± 1.9	-
M9-0.27 H-25LP	43	0.73%	20.6	± 1.5	35.0	± 1.7	-
M10-0.27 H-35LP	55	0.5%	28.5	± 1.6	42.3	± 1.8	-
M11-0.27 H-2SF	23	0.2%	29.3	± 1.1	40.8	± 1.3	0.75%
M12-0.27 H-6SF	25	0.14%	38.3	± 1.2	52.2	± 1.4	0.86%
M13-0.27 H-10SF	26	0.1%	43.1	± 1.3	55.0	± 1.6	1.06%
ACI/PTI 320 [16]	5–30 s	≤ 0.3%	≥ 21 MPa		≥ 35 MPa		+ 0.2%

### Effect of water to cementitious materials ratio

#### Flowability

As shown in Fig. 4, the control group mixes demonstrate a strong relationship between the water-to-cementitious materials ratio (W/Cm) and grout flowability. Higher W/Cm ratios, like in Mix M1-0.45-0, resulted in better flowability (efflux time of 12 s), while lower ratios led to increased viscosity and reduced flow. Mixes M2-0.35-0 and M3-0.27-0 both exceeded the acceptable efflux time limit, with M3 failing entirely due to the lack of chemical admixtures. The addition of a high-range water-reducing admixture (HRWR) in Mix M4-0.27 H-0 improved flow compared to M3, but the efflux time (39 s) still exceeded the standard threshold, indicating the HRWR dosage was insufficient to fully restore workability at such a low W/Cm ratio. This findings consists with Neville^25^ findings who explained that reducing water content increases mix viscosity, and HRWRs are required to achieve flowability, especially when targeting high-strength or low-bleed grouts.

#### Bleeding test

As presented in Fig. 5, bleeding performance in the control group closely correlated with the water-to-cementitious materials ratio (W/Cm). The highest W/Cm mix (0.45) showed excessive bleeding (3.2%), which is above the 0.3% limit. Reducing W/Cm to 0.35 lowered bleeding to 1.5% but remained non-compliant. A very low W/Cm of 0.27 without additives eliminated bleeding but caused poor flowability due to high viscosity. Adding HRWR at the same low W/Cm improved flow but still resulted in 1.0% bleeding. Overall, lower W/Cm improves bleeding resistance but reduces workability, while chemical admixtures help flowability but don’t fully control bleeding in low W/Cm mixes. This result was also confirmed by Neville^25^.

#### Compressive strength

Figures 6 and 7 show the impact of water-to-cementitious materials ratio (W/Cm) on compressive strength for control mixes at 7 and 28 days. At 7 days, higher W/Cm (0.45) in M1-0.45-0 yielded just 21.6 MPa, barely above the 21 MPa threshold. Lowering W/Cm to 0.35 (M2-0.35-0) slightly improved strength to 22.8 MPa. Ultra-low W/Cm of 0.27 without HRWR (M3-0.27-0) drastically increased strength to 49.9 MPa by reducing porosity and enhancing hydration. Adding HRWR at 0.27 W/Cm (M4-0.27 H-0) improved workability but resulted in slightly lower strength (40.1 MPa). At 28 days, all mixes gained strength: M1-0.45-0 reached 31.6 MPa but remained below the 35 MPa target; M2-0.35-0 surpassed it at 38.1 MPa. M3-0.27-0 showed the highest strength at 61.3 MPa, while M4-0.27 H-0 maintained good strength (57.1 MPa) with better workability, confirming that lower W/Cm ratios significantly enhance strength development. These results was also confirmed by several researchers^2,25^.

### Effect of fly ash

#### Flowability

As presented in Fig. 4, The Fly Ash Group mixes showed excellent flowability, with efflux times between 15 and 18 s which is within the standard limit (≤ 30 s). Mix M6-0.27 H-35FA performed best due to the spherical shape of fly ash particles, which reduced friction and improved flow. Mix M5-0.27 H-25FA had slightly lower flowability but still exceeded the Control Group’s performance, highlighting the positive impact of fly ash on grout rheology and workability. This also was confirmed by several researchers^2,25,26^.

#### Bleeding

As shown in Fig. 5, the Fly Ash Group demonstrated improved bleeding resistance compared to the Control Group. Mix M5-0.27 H-25FA exhibited a bleeding rate of 1.08%, while Mix M6-0.27 H-35FA recorded 0.33%. Although both values exceeded the BS PTI M55 threshold of 0.3%, the reduction is notable. The improved performance is attributed to the pozzolanic activity of fly ash, which reacts with calcium hydroxide and helps bind free water, thereby minimizing separation. The higher bleeding observed in M5 suggests that a 25% fly ash replacement was not sufficient to fully control water migration. In contrast, M6’s near-compliant bleeding rate underscores the dose-dependent effectiveness of fly ash in enhancing cohesion and reducing bleeding in grout systems. These findings coincide with several researchers findings^2,26^.

#### Compressive strength

Fly ash mixes demonstrated superior early and long-term compressive strength, driven by pozzolanic reactivity and microstructural refinement. At 7 days, M5-0.27 H-25FA and M6-0.27 H-35FA achieved strengths of 41.2 MPa and 35.3 MPa, respectively, significantly outperforming control mix M2-0.35-0 at 22.8 MPa. These highlights fly ash’s ability to enhance early-age strength despite its slower initial reaction compared to cement. By 28 days, these mixes showed sustained strength gains, with M5-0.27 H-25FA reaching 60 MPa and M6-0.27 H-35FA attaining 64 MPa, as shown in Fig. 7. This improvement results from the ongoing formation of C-S-H gel through the pozzolanic reaction between fly ash and calcium hydroxide, which refines the pore structure and densifies the matrix. This findings also matches findings of several researchers^2,26^.

### Effect of silica fume

#### Flowability

As illustrated in Fig. 4, grout mixes containing silica fume (SF) demonstrated moderate to excellent flowability, despite the material’s ultrafine particle size and high surface area, which typically increase mix viscosity. Mix M11-0.27 H-2SF (2% SF) achieved an efflux time of 23 s, while M12-0.27 H-6SF (6% SF) and M13-0.27 H-10SF (10% SF) recorded slightly higher values of 25 and 26 s, respectively. All mixes remained within the PTI M55.1-12 acceptable limit of ≤ 30 s, indicating sufficient workability. The incorporation of Sika ViscoCrete 3425 (HRWR) was instrumental in dispersing the fine SF particles, effectively reducing inter-particle friction and mitigating the viscosity increase typically associated with higher SF contents. Moderate SF dosages, such as 6%, appear to offer an optimal balance between enhanced packing density and acceptable flowability. Although higher SF content (10%) approaches the upper limit of compliance, it remains workable when used in conjunction with an effective HRWR dosage. Overall, the spherical morphology of SF contributes positively to particle packing, but its high surface area necessitates the use of superplasticizers to maintain flow performance. This also was confirmed by Rafat et al.^27^.

#### Bleeding

As shown in Fig. 5, all Silica Fume (SF) mixes met the ≤ 0.3% bleeding threshold, demonstrating excellent stability suitable for post-tensioning applications. Mix M11-0.27 H-2SF (2% SF) recorded a bleeding rate of 0.216%, while M12-0.27 H-6SF (6% SF) and M13-0.27 H-10SF (10% SF) achieved even lower rates of 0.137% and 0.107%, respectively. The improved performance with increasing SF content is attributed to the ultrafine particles’ ability to refine packing, absorb free water, and chemically bind water through pozzolanic reactions. This combination reduces capillary pores and restricts bleeding pathways. Figure 5 illustrates a clear trend of decreasing bleeding with higher SF dosages, highlighting SF’s dual function as a physical filler and chemical stabilizer which is unlike inert fillers such as limestone powder, which may increase bleeding at higher contents. This was also similar to Rafat et al.^27,28^findings.

#### Compressive strength

The Silica Fume (SF) Group comprises three grout mixes (M11-0.27 H-2SF, M12-0.27 H-6SF, and M13-0.27 H-10SF), which demonstrate exceptional compressive strength due to SF’s combined pozzolanic reactivity and filler effect. At 7 days, as shown in Fig. 6, all SF mixes met or exceeded the 21 MPa threshold, with strength increasing proportionally to SF dosage. M11-0.27 H-2SF (2% SF) achieved 29.25 MPa, primarily driven by improved particle packing and partial pozzolanic reaction. M12-0.27 H-6SF (6% SF) increased to 38.8 MPa due to enhanced reactivity, while M13-0.27 H-10SF (10% SF) reached the highest 7-day strength of 43.1 MPa, benefiting from SF’s high surface area and strong pozzolanic activity. By 28 days (Fig. 7), all SF mixes consistently exceeded the 35 MPa requirement, with further strength gains from ongoing pozzolanic reactions. M11-0.27 H-2SF attained 48.9 MPa, M12-0.27 H-6SF reached 52.2 MPa, and M13-0.27 H-10SF achieved 55 MPa, illustrating SF’s effectiveness in refining the microstructure and producing additional C-S-H gel. This was also found by Rafat et al.^27,28^.

#### Dimensional stability

As shown in Fig. 8, the volume change test was conducted on the control mix (M1-0.45-0) and selected mixtures that met compliance criteria in earlier tests, such as compressive strength, bleeding and flowability tests. As shown in Fig. 8, the control mix exhibited an expansion of 2.5%, exceeding permissible limits for dimensional stability, though no shrinkage was observed. This highlights the inherent instability of unmodified grouts with high water-to-cementitious ratios (W/Cm = 0.45). In contrast, silica fume (SF) mixes (M11-0.27 H-2SF, M12-0.27 H-6SF, M13-0.27 H-10SF) also showed expansion without shrinkage, attributed to the presence of Sika Intraplast Z, an expansive agent that counteracts drying shrinkage. Expansion increased with SF dosage, with the 10% SF mix (M13-0.27 H-10SF) showing the highest expansion and the 2% SF mix (M11-0.27 H-2SF) the lowest. This trend reflects SF’s role in refining microstructure and accelerating hydration in low-W/Cm systems. While SF mixes demonstrated controlled expansion within acceptable limits, the excessive deformation of the control mix underscores the risks of unmodified high-W/Cm formulations and highlights SF’s dual benefits in enhancing both durability and dimensional stability. This also was found by Neville et al.^29^.

### Effect of limestone powder

#### Flowability

Grout mixes containing limestone powder (LP) showed significantly reduced flowability as shown in Fig. 4 due to increased viscosity and inter-particle friction. Higher LP content consistently led to longer efflux times, with Mix M10-0.27 H-35LP reaching 55 s which is above the acceptable limit of 30 s. Even mixes with higher water content, like M7-0.30 H-25LP and M8-0.30 H-35LP, exceeded the flowability threshold. The angular shape of LP particles, acting as inert fillers, hindered particle movement and increased resistance. These results highlight that while LP may improve packing density, excessive use can severely impair workability, especially in low water-to-cementitious material ratio systems. These findings was also mentioned by Sua-iam et al.^30^.

#### Bleeding

The limestone powder (LP) group exhibited higher bleeding rates than mixes with pozzolanic additives, as shown in Fig. 5. Bleeding ranged from 1.4% in M7-0.30 H-25LP to 0.5% in M10-0.27 H-35LP, exceeding the PTI M55 limit of 0.3%. The reduction in bleeding with higher LP content and lower W/Cm ratio indicates that finer particle packing partially restricted capillary water channels. However, unlike reactive SCMs such as silica fume or fly ash, limestone powder lacks pozzolanic reactivity and does not bind free water chemically, resulting in residual water migration.The inert and angular nature of LP particles likely increased inter-particle friction, promoting localized segregation in mixes with higher water content (M7 and M8). Similar behavior was reported by Sua-iam and Makul^30^., who found that limestone acts primarily as a physical filler that enhances density but cannot prevent bleeding entirely. In contrast, silica fume reduced bleeding to below 0.2%, confirming the importance of chemical water binding and microstructural refinement in achieving durable, low-bleed grout.These results suggest that while LP can reduce bleeding compared to conventional grout, it should be combined with reactive SCMs or chemical stabilizers to achieve high-performance standards.

#### Compressive strength

The Limestone Powder (LP) Group consists of four grout mixes (M7-0.30 H-25LP, M8-0.30 H-35LP, M9-0.27 H-25LP, and M10-0.27 H-35LP), where compressive strength is influenced by both LP dosage and W/Cm ratio. At 7 days, as shown in Fig. 6, most mixes met or exceeded the 21 MPa threshold. M7-0.30 H-25LP achieved 24.7 MPa, and M8-0.30 H-35LP reached 28.8 MPa, benefiting from moderate to high LP content that improved packing density. In contrast, M9-0.27 H-25LP recorded 20.6 MPa, slightly below the threshold, due to the inert filler effect of LP limiting hydration efficiency despite the low W/Cm ratio. M10-0.27 H-35LP showed 28.5 MPa, reflecting improved particle packing but lacking the pozzolanic reactivity needed to generate additional C-S-H gel. By 28 days, significant strength gains were observed (Fig. 7). M7-0.30 H-25LP (24.7 MPa) and M8-0.30 H-35LP (29 MPa) remained below the 35 MPa target due to persistent porosity and the inert behavior of LP at the higher W/Cm ratio. Meanwhile, M9-0.27 H-25LP (35 MPa) and M10-0.27 H-35LP (42.3 MPa) met or exceeded the threshold, attributed to the low W/Cm ratio reducing capillary porosity and the role of LP particles as nucleation sites enhancing hydration. These findings also match with Sua-iam et al.^30^.

### Comparison with previous studies

The influence of W/Cm and HRWR on flowability and strength in our control and HRWR-modified mixes aligns qualitatively with Neville^25^ and Mohan et al.^15,18^, who reported that lowering W/Cm substantially increases compressive strength while reducing free flow unless rheology-modifying admixtures are used. Where our results differ numerically (for example, M4-0.27 H-0 giving 39 s efflux time versus some pre-blended grouts in Mohan et al. achieving lower efflux times at similar W/Cm), we attribute the difference to the type and dosage of HRWR used. Pre-blended commercial grouts frequently use optimized admixture packages (including viscosity-modifying agents and tailored superplasticizers) that are specifically designed to preserve flowability at low W/Cm; our site-batched approach used a simpler admixture system which explains the larger efflux times.

Second, the bleeding behavior we observed for silica fume and fly ash mixes concurs with Khayat et al.^8,9^ and Rafat et al.^27^, who showed that ultrafine, pozzolanic materials (silica fume) reduce bleed by both physical packing and chemical consumption/binding of free water. Our silica fume mixes reduced bleeding to well below the 0.3% limit, consistent with these studies. In contrast, the partial compliance of high-volume fly ash mixes (M6 close to the threshold) matches Mohan et al.^15^, where fly ash improves cohesion but may require higher replacement or complementary admixtures to fully suppress bleed. The intermediate performance of limestone powder which resulted in reduction in bleeding relative to plain grout but not to the level of SF supports the filler-only role of LP reported by Sua-iam & Makul^30^ and Randell et al.^13^. Importantly, variations in LP performance between our results and some literature reports likely reflect differences in LP particle size distribution and mineralogy.

Third, on compressive strength, our results show that low W/Cm mixes without excessive inert replacement deliver high strengths (e.g., M3 and M4). The strength gains for fly ash mixes at 28 days (M5–M6) are consistent with the well-known pozzolanic densification noted by Siddique^26^ and Mohan et al.^15^, where long-term strength exceeds that of control mixes. Where our fly ash mixes achieve somewhat higher long-term strengths than some published site-batched mixes, this can be explained by (i) the relatively low W/Cm used in our fly ash mixes together with HRWR, and (ii) good quality of the Class F fly ash used in our study. Conversely, mixes with high LP content showed lower early reactivity and sometimes reduced 7-day strengths. This behavior reported also by Sua-iam & Makul^30^. However, when combined with very low W/Cm (M10) filler effect partly compensated by 28 days, producing acceptable strengths. This underlines that LP can be used as a cost reducer provided the mix design targets sufficiently low W/Cm and adequate chemical admixture strategy.

Finally, regarding dimensional stability and expansion, our observation of controlled expansion in silica fume mixes (and excessive expansion in the high W/Cm control) agrees with other studies that emphasize the role of expansive admixtures and particle packing in early-age volume change (Neville & Aïtcin^29^. Some published studies report slight shrinkage rather than expansion for similar SF contents; the expansion in our SF mixes is therefore primarily linked to the presence and dosage of Sika Intraplast Z used in the study. This demonstrates that dimensional behavior cannot be explained by SCM type alone. The interaction of SCMs with expansive agents and curing regime is important to consider.

## Summary and conclusions

This research focused on developing an improved grout mix for post-tensioning systems that addresses common durability issues such as excessive bleeding, poor flowability, and shrinkage. Conventional grouts often suffer from water separation during curing and insufficient flow, which can lead to voids, corrosion risks, and incomplete tendon coverage. To overcome these challenges, grout mixes were developed using fly ash, limestone powder, silica fume, and chemical additives like Sika Intraplast Z and Sika ViscoCrete 3425. The grout mixes were tested for flowability, bleeding, compressive strength, and dimensional stability, aiming to achieve high performance and cost-effectiveness. While limestone powder alone cannot replace pozzolanic materials such as silica fume or fly ash in achieving high-performance grout standards, its use remains beneficial in practical contexts. As a locally sourced, low-cost material, it can serve as a partial replacement to reduce production costs and environmental impact, especially in regions with limited access to advanced SCMs. When used in combination with reactive additives, limestone powder can contribute to improved bleeding resistance and reduced material costs without severely compromising grout performance. The conclusions of the study are as follows:

1. Reducing the water-to-cement (W/C) ratio in conventional grout mixes significantly enhances compressive strength and reduces bleeding. However, the reduction in W/C ratio also leads to a considerable decrease in flowability, falling below the standard limits required for high-performance grout applications.
2. Incorporating a high-range water-reducing admixture (Sika ViscoCrete 3425) into grout mixes with a low W/C ratio of 0.27 considerably improves flowability. However, this results in a slight compromise in compressive strength and an increase in bleeding. Despite these improvements, the mixes still fall short of meeting the high-performance grout standards for both flowability and bleeding.
3. Replacing 25% of cement with fly ash significantly enhances the workability of low W/Cm grout mixes, enabling compliance with flowability standards, and notably improves compressive strength. However, the bleeding rate remains above the permissible limits.
4. Increasing fly ash content to 35% further improves all grout properties such as flowability, compressive strength, and bleeding and brings the mix close to achieving all high-performance grout requirements. Nevertheless, the high cost associated with fly ash and its elevated replacement level limit its widespread practical use.
5. Limestone powder (LP) acts primarily as an inert filler rather than a reactive mineral admixture. While increasing LP content helps reduce bleeding, it also leads to declines in both compressive strength and flowability, thereby limiting its effectiveness in high-performance grout mixes.
6. Introducing silica fume into grout mixes yielded the best overall performance with only a marginal increase in cost. Silica fume mixes successfully met the standard requirements for high-performance grout, offering an optimal balance of workability, strength, and bleeding resistance.
7. Increasing the silica fume replacement percentage results in a slight reduction in flowability, though values remain within acceptable limits. Simultaneously, bleeding is further reduced, and compressive strength is enhanced, confirming the effectiveness of silica fume as a high-performance grout component.

## Data Availability

The data will be made available by the authors upon reasonable request. Please contact the corresponding author, Dr. Ayman Shamseldein, for access.
